# Vortex Glass—Vortex Liquid Transition in BaFe_2_(As_1-x_P_x_)_2_ and CaKFe_4_As_4_ Superconductors from Multi-Harmonic AC Magnetic Susceptibility Studies

**DOI:** 10.3390/ijms24097896

**Published:** 2023-04-26

**Authors:** Ion Ivan, Alina M. Ionescu, Daniel N. Crisan, Adrian Crisan

**Affiliations:** National Institute of Materials Physics, 405A Atomistilor Str., 077125 Magurele, Romania; ion.ivan@infim.ro (I.I.); alina.ionescu@infim.ro (A.M.I.); daniel.crisan@infim.ro (D.N.C.)

**Keywords:** vortex glass, Lindemann melting, anisotropic superconductors, pnictides, vortex melting line, multi-harmonic susceptibility

## Abstract

For practical applications of superconductors, understanding the vortex matter and dynamics is of paramount importance. An important issue in this context is the transition of the vortex glass, which is a true superconducting phase, into a vortex liquid phase having a linear dissipation. By using multi-harmonic susceptibility studies, we have investigated the vortex glass—vortex liquid phase transitions in CaKFe_4_As_4_ and BaFe_2_(As_0.68_P_0.32_)_2_ single crystals. The principle of our method relates the on-set of the third-harmonic susceptibility response with the appearance of a vortex-glass phase in which the dissipation is non-linear. Similar to the high-critical temperature cuprate superconductors, we have shown that even in these iron-based superconductors with significant lower critical temperatures, such phase transition can be treated as a melting in the sense of Lindemann’s approach, considering an anisotropic Ginzburg-Landau model. The experimental data are consistent with a temperature-dependent London penetration depth given by a 3D *XY* fluctuations model. The fitting parameters allowed us to extrapolate the vortex melting lines down to the temperature of liquid hydrogen, and such extrapolation showed that CaKFe_4_As_4_ is a very promising superconducting material for high field applications in liquid hydrogen, with a melting field at 20 K of the order of 100 T.

## 1. Introduction

The discovery of superconductivity in cuprates at much higher critical temperatures *T*_c_ than in the classical superconductors (metals, inter-metallic compounds or alloys) raised the fundamental question regarding the influence of thermally-activated processes in these new materials, given the much larger thermal energy *k*_B_*T*. It has long been recognized that defects (disorder) that act as flux pinning centers in the mixed state of type-II superconductors destroy the translational long-ranged order (TLRO) of the Abrikosov flux lattice [1] and lead to an extremely low voltage due to vortex creep at small currents [2]. Since disorder destroys the TLRO, the question of the existence of a sharp equilibrium phase boundary separating the normal phase at low temperatures and magnetic fields *H* (but *H* larger than the lower critical field *H*_c1_) arises. The original Anderson-Kim theory [2], although predicting a strong crossover from fast to slow dynamics upon cooling, gives no sharp phase boundary since. Even at low *T*, a nonzero (flux flow) resistance is predicted, as in the normal phase (but obviously having a much smaller value). However, in a seminal paper [3], M.P.A Fisher argued that in bulk disordered systems there is a sharp equilibrium phase boundary below which exists a new thermodynamic phase, namely a vortex-glass (VG) superconducting state. In certain systems (granular superconductors) and in certain conditions, mean-field theoretical treatment led to a glass phase similar to an *XY* spin-glass [4]. Fisher, Fisher, and Huse [5] provided a consistent and comprehensive theoretical treatment, backed by various experimental proofs, on the thermal fluctuations, quenched disorder, phase transitions, and transport in type-II superconductors. They argued that a vortex-glass phase may occur with vanishing resistivity and long-range phase coherence, with a nontrivial spatial structure reflecting the positions of the randomly pinned vortices. This ordering into a specific nontrivial arrangement determined by the particular details of the quenched disorder in the system is very analogous to the magnetic order that occurs in a spin glass (hence the name vortex glass). The vortex glass phase is most conveniently described through its response: as the current *J* goes to zero the barriers inhibiting vortex motion *U* diverge as *U*_c_(*J*_c_/*J*)^µ^, and the vortex velocity *v* ∝ exp[−*U*(*J*)/*T*] goes to zero in a singular manner (*U*_c_ and *J*_c_ represent some characteristic values of the barrier and current). The glass exponent µ describing the divergence of the barriers is a characteristic quantity of the vortex phase. As the temperature is raised, increased thermal fluctuations will cause the vortex glass phase to disorder (melt) into a vortex fluid/liquid (VL) phase at a temperature lower than the mean-field transition, wherein the superconductor becomes normal. The vortex liquid phase is a fully disordered phase that is not separated from the normal phase by a true phase transition. They should be connected by a smooth cross-over, the true phase transition being the one between the vortex glass and the vortex fluid at a temperature-dependent melting field *B*_M_(*T*).

A decade later, C. Reichhardt and co-authors [6] suggested that in some systems vortices freeze in the same way as window glass, and introduced the vortex molasses (VM) scenario based on numerical simulations (the main difference being the dependence of resistivity at the transition). Their work was ignited by some experimental results on un-twinned YBa_2_Cu_3_O_7-x_ single crystals, very “clean” thus without strong pinning centers. Our samples are known to have a large density of strong pinning centers and, in addition, in both VG and the newly-proposed VM, the dissipation is non-linear, so our method stands.

The VG-VL melting transition can be treated using the Lindemann melting approach, where the thermally driven disorder of the vortex solid (lattice, or glass in the presence of quenched disorder) can be quantified by the Lindemann index, which is a mean-square displacement of a vortex line in a system composed of *N* vortices per unit surface perpendicular to the applied field with separation between them being *r_ij_* (*i*, *j* from 1 to *N*, *i* ≠ *j*):

(1) $$q_{i}=\frac{1}{N-1}\sum_{j\neq i}\frac{\sqrt{\langle r_{ij}^{2}\rangle -{\langle r_{ij}\rangle }^{2}}}{\langle r_{ij}\rangle }$$

where angle brackets indicate a time average. The global Lindemann index *q* is a system average, and the system melting is considered to occur at a certain temperature where *q* deviates from linearity, or increases above a threshold value, which in the classical, empirical theory of melting is taken as *c_L_a*_0,_ with *a*_0_ being the lattice spacing (or the average distance between near-neighbors in glasses) and *c_L_* is the Lindemann number (usually chosen to be a constant between 0.1 and 0.3). In the case of high-temperature superconductors, *c*_L_ ≈ 0.12–0.15 gave results consistent with various experiments, and, most importantly, the “lattice spacing” depends on the applied magnetic field *B* as *a*_0_ ≈ (ϕ_0_/*B*)^1/2^, with ϕ_0_ being the magnetic flux quanta.

The fact that the vortex glass melting line in the temperature-field space diagram separates the true superconducting phase (suitable for practical applications) from the dissipative liquid phase makes this line a paramount property of various superconducting materials. The aim of this work is to determine the vortex glass—vortex liquid melting line in single crystals of two of the most important iron-based superconductors, discovered quite recently, by using a straight-forward method that implies multi-harmonic susceptibility response of the material to an AC magnetic field excitation superimposed on a high DC magnetic field, an original method developed first for Tl-based high-temperature cuprate superconductors [7]. To our knowledge, such measurements were not described so far for iron-based superconductors, so this work also aims at checking if our method is suitable for iron-based superconductors.

## 2. Materials and Methods

CaKFe_4_As_4_ single crystals were synthesized in AIST Tsukuba, Japan, via the self-flux method using FeAs, the preparation being described in detail in [8]; they are thin square discs, having the typical dimensions *a* ≈ 1.2 mm, *b* ≈ 1.2 mm and *c* ≈ 0.04 mm. The BaFe_2_(As_0.68_P_0.32_)_2_ single crystals analysed in this work have been grown by the Ba_2_As_3_/Ba_2_P_3_-flux method [9] at the Institute of Physics, Chinese Academy of Sciences; they are also square-shaped, with dimensions *a* ≈ 1.7 mm, *b* ≈ 1.7 mm and *c* ≈ 0.05 mm. Depending on the growth conditions, the amount of P in the BaFe_2_(As_1-x_P_x_) single crystals can be between x = 0.21 (heavily under-doped) and x = 0.64 (heavily over-doped). It was shown that the optimum doping, resulting in the largest critical temperature of 29 K, is with P content corresponding to x = 0.3. BaFe_2_(As_0.68_P_0.32_)_2_ sample studied here is a slightly over-doped sample with a critical temperature of 27.8 K.

The multi-harmonic AC susceptibility studies were performed using a commercial *Quantum Design* Physical Property Measurement System (PPMS), with frequencies up to 10 kHz and AC field amplitudes up to 16 Oe, in DC fields up to 14 T in the case of CaKFe_4_As_4_ single crystal, or up to 7 T in the case of BaFe_2_(As_0.68_P_0.32_)_2_ single crystal. In all of the experiments reported here, the applied field (both DC and AC) is parallel to the *c*-axis (perpendicular to the largest plane *a*–*b*) of the single crystals.

## 3. Results

The principle of our method comes from the very basic properties of vortex matter described in the introduction: in the VG state below the field-dependent melting temperature *T_m_*(*B*), the electric field response (dissipation) to a current density *J* is strongly nonlinear, of the form *E*(*J*) ~ exp[−(*J_T_*/*J*)^µ^], where *J_T_* is a characteristic current density and µ ≤ 1. Exactly at *T_m_*(*B*) the current-voltage characteristic is a power law, and, finally, for *T* > *T_m_*(*B*) (in the VL state), there is an ohmic behavior *E*(*J*) ~ *J* for sufficient low current levels. The first method to determine the VG-VL transition was through measurements of current-voltage (*I-V*) characteristics at extremely low dissipation levels, usually by using a very sensitive pico-voltmeter, at a large number of temperatures around the melting temperature *T*_m_ in a fixed DC applied field. Double-logarithmic plot of *I-V* curves shows downward curvatures for temperatures smaller than *T*_m_, upward curvatures for temperatures higher than *T*_m_ (at low voltage levels), and a straight line (in the double logarithmic plot), i.e., a power-law relation of the form *V*(*I*, *T* = *T*_m_) ∝ *I*^(*z*+1)/(*d*−1)^ where *z* is the dynamical exponent of the VG and *d* is the dimensionality [5]. However, this method is very difficult to employ, since it requires ultra-sensitive voltmeter and exceptional contacts with very low resistivity for the transport measurements. For these reasons, contact-less magnetic measurements are highly desirable. In a solid sample immersed in a magnetic field having the strength *H* there will be a magnetic flux density *B*. If the solid has also magnetic properties, there will be the sample magnetization *M* = χ*H*, where χ is the magnetic susceptibility of the sample, so magnetic flux density will be *B* = µ_0_(1 + χ)*H* with µ_0_ being the magnetic permeability constant. If the magnetic field is a periodic function, then magnetization can be expressed in a Fourier expansion. For a function *f* which is periodic with the period 2π, Fourier expansion is:

(2) $$f\left(t\right)=\frac{1}{2}a_{0}+\sum_{n=1}^{\infty }\left[a_{n}\cos nt+b_{n}\sin nt\right],$$

where $a_{n}=\frac{1}{\pi }\int_{-\pi }^{\pi }f\left(t\right)\cos\;nt\;dt$ and $b_{n}=\frac{1}{\pi }\int_{-\pi }^{\pi }f\left(t\right)\sin\;nt\;dt$ are the Fourier coefficients.

**Figure 1:**
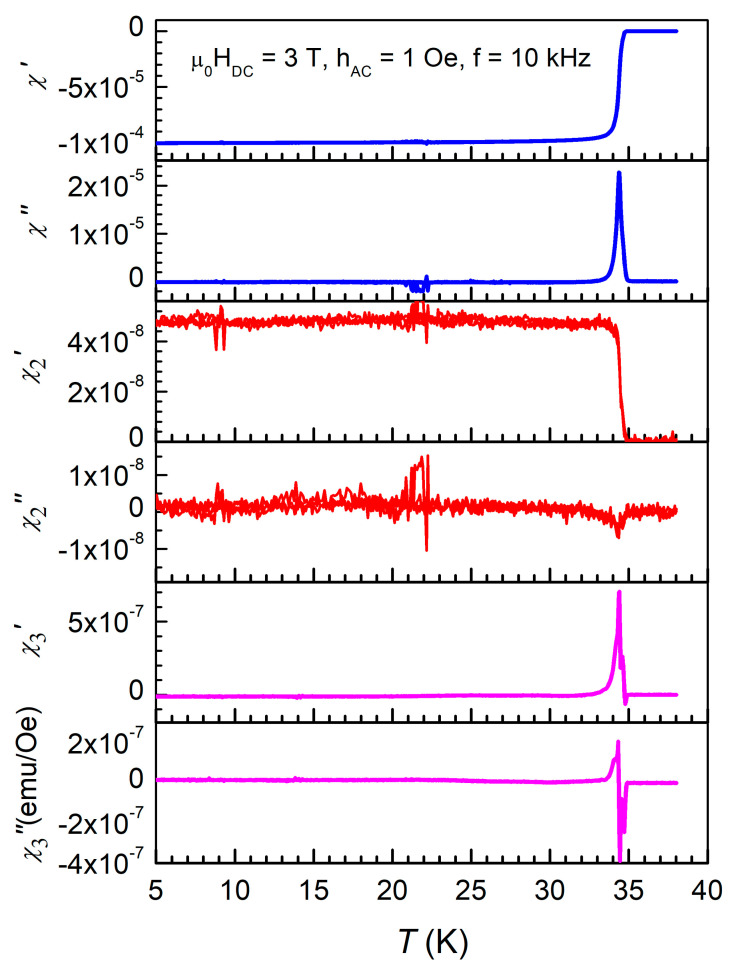
Temperature dependence of in-phase (χ’) and out of phase (χ”) susceptibility of CaKFe_4_As_4_, from top to bottom: fundamental (*n* = 1), second harmonic (*n* = 2) and third harmonic (*n* = 3), displayed in emu/Oe, measured in the conditions shown in the figure (µ_0_*H*_DC_ = 3 T, *h*_AC_ = 1 Oe, *f* = 10 kHz).

In our case, the Fourier expansion of the time-dependent magnetization *M*(2*πf*_1_*t*) of the sample, in a magnetic field *H*(2*πf*_1_*t*) = *H*_DC_ + *h*_AC_cos(2*πf*_1_*t*) takes the form

(3) $$M\left(2\pi f_{1}t\right)=\chi_{0}H_{DC}+h_{AC}\sum_{n}\left[\chi_{n}^{\prime }cos\left(2\pi nf_{1}t\right)+\chi_{n}^{\prime \prime }sin\left(2\pi nf_{1}t\right)\right]$$

where *f*_1_ is the fundamental frequency of the excitation AC magnetic field while χ’_n_ and χ”_n_ are the in-phase and out-of-phase components of the harmonic susceptibility, with *n* = 1, 2, 3, etc., and represents the Fourier coefficients of the magnetization. The first term in Equation (3), χ_0_*H*_DC_ is an offset by the DC magnetic field. Figure 1 shows the temperature dependence of the terms of the complex susceptibility of the CaKFe_4_As_4_ single crystal from Equation (3), χ’_n_ and χ”_n_, for *n* = 1, 2, and 3, in a DC field of 3 T, measured with an excitation AC field with amplitude of 1 Oe and frequency of 10 kHz.

In the first panel, representing the fundamental in-phase susceptibility response, a very sharp superconducting transition can be seen, with a diamagnetic response of about 10^−4^ emu/Oe, practically constant just below the critical temperature *T*_c_, the sample entering the superconducting state which is generated by the shielding supercurrents. The fundamental out-of-phase susceptibility has a sharp peak around *T*_c_, with a peak value of about 2 × 10^−5^ emu/Oe, and is proportional to the dissipation. The in-phase second harmonic is almost constant below *T*_c_, 4 × 10^−8^ emu/Oe, while the out-of-phase second harmonic is practically just noise. The in-phase and out-of-phase third harmonic susceptibility display sharp features near *T*_c_, in the range of 5 × 10^−7^ emu/Oe. Fabbricatore et al. [10] performed a comprehensive theoretical and experimental work on the effects of vortex dynamics on higher harmonics of AC susceptibility in type-II superconductors. For the purpose of our study, the most important result of their work is that the odd harmonics (e.g., third harmonics in our case) is generated only by nonlinearity in the current-voltage curves which means that, if the out-of-phase fundamental susceptibility is the measure of the total dissipation, the third harmonic susceptibility is the measure of the ***nonlinear dissipation only***. The even harmonic (second harmonic in Figure 1) is a measure of non-symmetries, which may arise if the DC field and AC excitation field are not perfectly parallel, if the single crystal’s *c*-axis is not perfectly parallel with the magnetic field(s), or if the single crystal itself is not symmetric.

**Figure 2:**
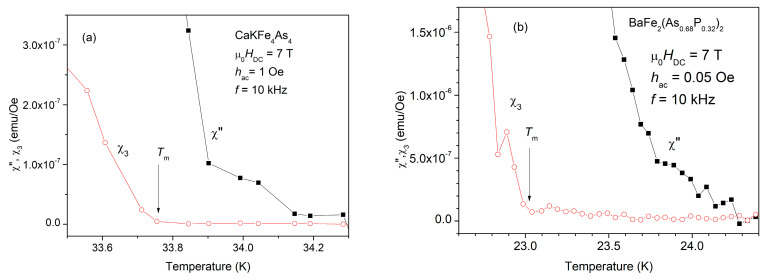
Temperature dependence of the out-of-phase fundamental susceptibility and of the module of the third harmonic, in a DC field of 7 T, near the transition temperature, zoomed at very low dissipation levels, for the: (**a**) CaKFe_4_As_4_ single crystal using an excitation field of amplitude 1 Oe and frequency 10 kHz; and (**b**) BaFe_2_(As_0.68_P_0.32_)_2_ single crystal using an excitation field of amplitude 0.05 Oe and frequency 10 kHz. A much smaller AC excitation field amplitude was used because this single crystal has a much smaller critical current density.

To distinguish between the on-set of the non-linear dissipation and the on-set of the total dissipation, one has to look very close to the transition temperature, and to perform a zoom very near those on-sets. Figure 2 shows such zoom for the two different single crystals, in a DC field of 7 T.

In both panels of Figure 2 it can be clearly seen that the on-set of the third harmonic susceptibility, marked with an arrow in the figure, appears at a temperature *T*_m_(*H*_DC_), field-dependent vortex melting temperature, lower than the on-set temperature of the fundamental out-of-phase susceptibility (which is actually the critical temperature *T*_c_(*H*_DC_) for the DC field of 7 T, or, in other words, 7 T is the upper critical field at the temperature where onset of χ” appears). It can also be seen that, at 7 T, the difference in the two temperatures, *T*_c_(*H*_DC_) − *T*_c_(*H*_m_) is smaller for the 1144 single-crystal (about 0.4 K) than for the 122 single crystal (>1 K).

**Figure 3:**
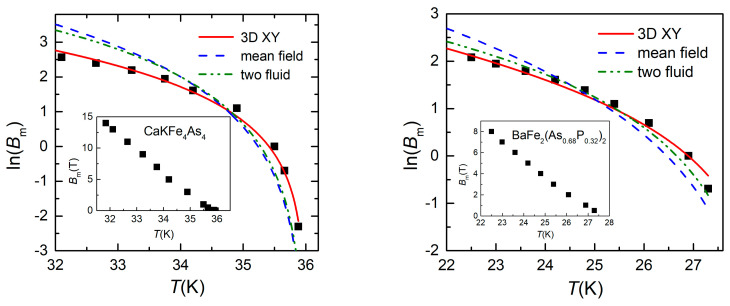
Temperature dependence of ln(*B*_m_) for the: (**a**) CaKFe_4_As_4_ single crystal; and (**b**) BaFe_2_(As_0.68_P_0.32_)_2_ single crystal. Lines represent the fit with the three models discussed in the following section. Inserts show the same experimental data presented as *B*_m_(*T*). Inserts show the experimental data in normal scale, melting field *B*_m_ as function of temperature *T*.

Between the two temperatures, *T*_m_(*H*_DC_) and *T*_c_(*H*_DC_) there is the vortex liquid phase, where there is only linear dissipation, while below *T*_m_(*H*_DC_) there is also non-linear dissipation, hence there is vortex glass. Repeating such measurements for many more applied DC fields, the vortex glass—vortex liquid melting line can be constructed, either as *T*_m_(*B*), with *B* = µ_0_*H*_DC_, or as *B*_m_(*T*). Inserts in Figure 3 show the *B*_m_(*T*) experimental points (full black squares) for the two single crystals, while the same symbols in the main panels represent the ln(*B*_m_) vs. *T* experimental data. The meaning of the lines in Figure 3 will be addressed in the next section.

## 4. Discussion

Analysis of the melting transition in type-II anisotropic superconductors, subjected to an applied DC field with arbitrary orientation, in the framework of an anisotropic three-dimensional (3D) Ginzburg-Landau rescaling approach [11] gives the following temperature dependence of the melting field:

(4) $$B_{m}\left(T\right)=\frac{C^{2}c_{L}^{4}\phi_{0}^{5}}{{\left(k_{B}T\right)}^{2}\lambda_{ab}^{4}\gamma {\left(cos^{2}\alpha +\gamma^{2}sin^{2}\alpha \right)}^{1/2}}\;,$$

where *C* ≈ 1/4π^2^ is a constant, *c_L_* is the empirical Lindemann parameter, ϕ_0_ is the magnetic flux quanta, λ*_ab_* is the penetration depth along the (*a*,*b*) plane, γ is the anisotropy factor, and *α* is the angle between the applied magnetic field lines and the (*a*,*b*) plane. For our single crystals and with the experimental setup used, *α* = π/2, cos*α* = 0, sin*α* = 1, and Equation (4) becomes

(5) $$B_{m}\left(T\right)=\frac{C^{2}c_{L}^{4}\phi_{0}^{5}}{{\left(k_{B}T\right)}^{2}\lambda_{ab}^{4}\gamma^{2}}\;$$

It can be seen that the melting field depends on temperature from two sources, the thermal activation energy *k*_B_*T*, and the temperature-dependent penetration depth λ*_ab_*. Regarding the temperature dependence of the in-plane penetration depth λ*_ab_*, there are currently three different models: (i) critical behavior of the 3D *XY* model [12] which predicts λ*_ab_*(*T*) = λ*_ab_*(0)(1 − *T*/*T*_c_)^−1/3^; (ii) mean-field approximation with λ*_ab_*(*T*) = λ*_ab_*(0)(1 − *T*/*T*_c_)^−1/2^; and (iii) two-fluid model [13] which predicts λ*_ab_*(*T*) = λ*_ab_*(0)[1 − (*T*/*T*_c_)^4^]^−1/2^. In the main panels of Figure 3 are shown the one-parameter fits of the experimental data with Equation (5), for the two single crystals: 3D *XY* model (red full lines), mean field model (blue dash lines), and two-fluid model (green dash dot lines). It can be clearly seen that the best fit is with the 3D *XY* model.

As mentioned before, such experiments and analysis were performed on cuprate superconductors, especially those grown by high-pressure synthesis (mostly metastable superconductors that cannot be produced as large single crystals or epitaxial thin films), for estimations of the anisotropy factors. The first work [7] studied the TlBa_2_Ca_2_Cu_3_O_10-y_, and, for comparison and proof-of-principle of the new technique, the well-known and most studied YBa_2_Cu_3_O_7-δ._ For both materials, the experimental results were best fitted (one-parameter fit) using the λ*_ab_*(*T*) dependence given by the critical behavior of the 3D *XY* model. However, later on it was found that the 3D *XY* model is not the best for all cuprates. A first example was provided by (Cu_0.6_C_0.4_)Ba_2_Ca_3_Cu_4_O_y_ [(Cu,C):1234] and (Cu_0.5_C_0.5_)Ba_2_Ca_2_Cu_3_O_y_ [(Cu,C):1223] multi-component cuprates having various carrier concentrations obtained by annealing in flowing nitrogen gas in various conditions. It was shown [14] that the vortex melting lines of all (Cu,C):1223 samples and of optimum-doped (Cu,C):1234 were well described by the two-fluid model. Overdoped (Cu,C):1234 proved to have an anomalous melting line which was explained by a significant opening of a second superconducting gap due to CuO_2_ outer planes at a temperature lower than the critical one, where the first superconducting gap due to inner planes opens. Two-fluid model was successful in describing the vortex melting lines of Hg-based multi-layer (*n* = 3, 4, 5) and super-multi-layer cuprates (*n* ≥ 6) HgBa_2_Ca*_n_*_−1_Cu_n_O_y_ [15], and in a series of cuprates with F-substitution at apical oxygen site, Ba_2_Ca_3_Cu_4_O_8_(O_1−y_F_y_)_2_ (2*y* = 2) and Ba_2_CaCu_2_O_4_(O_1−y_F_y_)_2_ (2*y* = 1.3, 1.6, 2) [16]. A further investigation of Ba_2_Ca_3_Cu_4_O_8_(O_1−y_F_y_)_2_ samples with different fluorine content [17] showed that the heavily underdoped sample (2*y* = 2) is described by the two-fluid model, as previously mentioned, the underdoped sample (2*y* = 1.6) is described by the mean-field model, while the near-optimally doped sample (2*y* = 1.3) is better described by the 3D *XY* model.

The one-parameter fit of the vortex melting line of CaKFe_4_As_4_ single crystal in Figure 3a with the temperature dependence of the penetration depth given by the 3D *XY* model was performed using a Lindemann parameter *c_L_* = 0.12, critical temperature *T*_c_ = 36 K (as resulted from our measurement), and λ*_ab_*(0) = 208 nm as determined from muon-spin rotation measurements [18], with the resulting fit parameter being the anisotropy factor γ = 2.11 in perfect agreement with other types of measurements reported in the literature. In the case of the similar fit of the vortex melting line of the BaFe_2_(As_0.68_P_0.32_)_2_ single crystal in Figure 3b, *c_L_* = 0.12, critical temperature *T*_c_ = 27.8 K, but the possible value of λ*_ab_*(0) needs further attention. The first results on the BaFe_2_(As_1-x_P_x_)_2_ system determined λ*_ab_*(0) = 108 nm [19], but most likely the samples studied and reported there were not very close to *x* = 0.3, where a work by Hashimoto et al. [20] demonstrated the existence of a sharp peak of λ*_ab_*(0), (≈300 nm) at optimum composition (*x* = 0.3) in BaFe_2_(As_1-x_P_x_)_2_, most likely due to the closeness of a Quantum Critical Point. Carefully studying the data in [8] allowed us to estimate a reasonable value for λ*_ab_*(0) in the case of our slightly over-doped single crystal (*x* = 0.32) to be around 250 nm. With this value, the resulted fitting parameter is γ = 4.5, a quite reasonable value.

**Figure 4:**
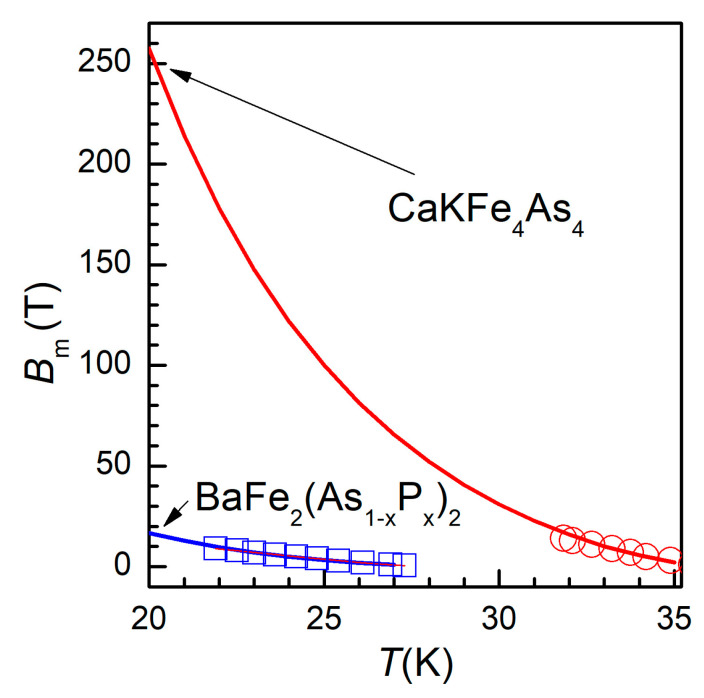
Temperature dependence of the vortex melting field *B*_m_, extrapolated to the temperature of liquid hydrogen, using the fitting parameters described in the text, for BaFe_2_(As_1−x_P_x_), blue squares, and of CaKFe_4_As_4_, red circles.

Let’s now extend our discussion to the possible practical applications of these new superconducting materials. Considering the increased interest in a clean, sustainable hydrogen-based economy, in which liquid hydrogen will be an abundant, cheap, energy vector, possible applications of superconducting materials at 20 K are becoming very attracting. Based on the experimental data and the best fits, we have extrapolated the vortex melting lines towards *T* = 20 K, using the fitting parameters described above, to have an idea about how large the melting (irreversibility) field may be at liquid hydrogen temperature. The extrapolation is shown in Figure 4, showing a huge *B*_m_(20 K) of about 250 T.

It can be clearly seen that, unlike BaFe_2_(As_0.68_P_0.32_)_2_ which has a critical temperature of 28.5 K and a reasonably high melting field at 20 K (<20 T), CaKFe_4_As_4_ is a very remarkable and promising material for high-field applications in liquid hydrogen, with a critical temperature of 36 K. However, a closer look at the main panel of Figure 3a shows that at lowest temperature the full red line goes above the experimental point, and, considering the range of extrapolation, more reasonable value of *B*_m_(20 K) is probably around 100 T, which is a very large value for most conceivable practical applications.

## 5. Conclusions

In conclusion, we have investigated the vortex glass—vortex liquid phase transitions in two representative single crystals from the 1144 and 122 families of newly-discovered iron-based superconductors, namely CaKFe_4_As_4_ and BaFe_2_(As_0.68_P_0.32_)_2_. We have employed for our studies multi-harmonic AS susceptibility measurements with the samples subjected to a superposition of high DC magnetic fields and AC excitation magnetic field, using commercial *Quantum Design* MPMS systems. The magnetization response of the samples in the periodic field was written as Fourier expansion, with the Fourier exponents being the in-phase and, respectively, out-of-phase multi-harmonic susceptibility. The MPMS system can record up to the 9th harmonic, but for this study we have employed only up to the third harmonic. The principle of our method is based on the fundamental properties of vortex glass, namely the strongly non-linear dissipation, unlike the linear dissipation that occurs in the vortex liquid phase. As for the susceptibility response, the out-of-phase fundamental susceptibility is a measure of total dissipation, linear plus non-linear, while the third harmonic is a measure of non-linear dissipation only. By determining the temperature where the on-set of the third harmonic occurs, at a given DC field, we were able to construct the vortex glass vortex liquid melting lines for the two single crystals. Theoretical and numerical analysis allowed us to conclude that our method is suitable also for iron-based superconductors; to determine that the temperature-dependence of the London penetration depth suitable for our samples is given by the model of 3D *XY* fluctuations; and to determine the anisotropy factors, which are in good agreement with other methods in the literature. From a practical point of view in terms of high-field applications at liquid hydrogen temperature, extrapolated values of the melting field are about 20 T for the 122 crystal, and of the order of 100 T, (considering the much larger domain of our speculative extrapolation, hence much larger errors), for the 1144 crystal.

## Data Availability

All of the data reported are available upon request.
